# Acute and Delayed Deaths after West Nile Virus Infection, Texas, USA, 2002–2012

**DOI:** 10.3201/eid2502.181250

**Published:** 2019-02

**Authors:** David C.E. Philpott, Melissa S. Nolan, Nicole Evert, Bonny Mayes, Dawn Hesalroad, Eric Fonken, Kristy O. Murray

**Affiliations:** Baylor College of Medicine and Texas Children’s Hospital, Houston, Texas, USA (D.C.E. Philpott, M.S. Nolan, K.O. Murray);; Texas Department of State Health Services, Austin, Texas, USA (N. Evert, B. Mayes, D. Hesalroad, E. Fonken)

**Keywords:** West Nile virus, viruses, WNV, West Nile neuroinvasive disease, WNND, meningitis/encephalitis, acute deaths, delayed deaths, standardized mortality ratios, hazard ratios, survival analysis, Texas, United States

## Abstract

Infection with West Nile virus (WNV) has a well-characterized acute disease process. However, long-term consequences are less understood. We searched death records for 4,142 residents of Texas, USA, infected with WNV during 2002–2012 and identified 557 (13%) deaths. We analyzed all-cause and cause-specific deaths after WNV infection by calculating standardized mortality ratios and using statewide mortality data. Acute-phase deaths (<90 days after symptom onset) occurred in 289 (7%) of case-patients; of those deaths, 289 (92%) were cases of West Nile neuroinvasive disease (WNND). Convalescent-phase deaths (>90 days after symptom onset) occurred in 268 (7%) of the remaining 3,853 case-patients; 210 (78%) of these deaths occurred in patients with WNND. Convalescent-phase WNND case-patients showed excess deaths from infectious and renal causes; case-patients <60 years of age had increased risk for all-cause death, specifically from renal, infectious, digestive, and circulatory causes. We provide population-level evidence of increased risk for death after WNV infection resulting in WNND.

## Abstract 2

Infected patients should be closely monitored for prevention of future health problems.

West Nile virus (WNV) is an arbovirus that can result in severe disease and death in humans. Since the first outbreak of infection with this virus in the United States during 1999, it has emerged across the country and resulted in >46,000 clinical cases of infection across all contiguous states (*1*). Uncomplicated febrile illness, known as West Nile fever (WNF), will develop in 20% of persons acutely infected, and severe West Nile neuroinvasive disease (WNND) will develop in <1% (*2*). Acute WNV infection can cause substantial long-term disability resulting in neurologic, neuropsychologic, and kidney disease outcomes; higher risks for poor outcome are associated with a diagnosis of WNND (*3**–**9*). In addition to continual illness, evidence from 2 small cohorts suggested that WNV can result in excessive deaths (*10**,**11*).

Death registry–based linkage studies on other virus diseases, such as hepatitis, have characterized the patterns and risk for death at the population level (*12**–**15*). We used a similar death registry–based method to describe specific causes of death for WNV case-patients to determine whether this population shows excess death years after initial onset. We analyzed acute-phase and convalescent-phase specific causes of death in WNV case-patients given a diagnosis in Texas, USA, during 2002–2012 to determine whether persons with WNV had an increased risk for death than the general population. Our study objectives were to 1) describe causes of death in our population and identify any potential covariates, with particular attention to age because it is a known risk factor for severe disease; 2) compare all-cause and cause-specific mortality rates with those of the underlying Texas population by using standardized mortality ratios (SMRs); and 3) examine yearly deaths for WNV-infected case-patients as they progress through time.

## Methods

### Study Population

In Texas, WNV infections are reported to the Texas Department of State Health Services (TXDSHS) by a passive surveillance system and coded by using standard case definitions for neuroinvasive and nonneuroinvasive diseases (*16*). WNND includes meningitis, encephalitis, meningoencephalitis, and acute flaccid paralysis; non-WNND includes only WNF. Asymptomatic viremic blood donors were not included in the available TXDSHS database. During July 3, 2002–December 6, 2012, a total of 4,162 WNV cases were reported to TXDSHS; 4,142 cases had complete data on age and US Social Security numbers and were included in our analysis.

We linked the WNV case dataset of TXDSHS to the Texas Death Registry by using Social Security numbers. All deaths identified through December 31, 2012 in the registry were included in this study, regardless of cause or timing of death. Death records were abstracted for age, sex, race, date of death, and underlying cause of death by the International Classification of Diseases, 10th Revision (ICD-10), code listed on the death certificate. Underlying cause of death is defined by the National Center for Health Statistics as “the disease or injury which initiated the train of morbid events leading directly to death or the circumstances of the accident or violence which produced the fatal injury” (*17*).

We chose underlying cause of death because it is commonly used in death studies and because it indicates what the clinician believed was the major cause of death, even accounting for other concurrent illnesses that might also have contributed to the death of a given individual. In addition, because data were incomplete for contributing and immediate causes of death, we were unable to obtain reliable estimates for these causes. Underlying causes of death were grouped according to ICD-10 chapters: infectious, renal, neoplasms, blood/immune, endocrine, nervous, circulatory, respiratory, digestive, genitourinary, other, and external forces. Twenty-four deaths did not have cause of death data and were excluded from specific cause of death analysis; however, all 24 of these persons died during first 88 days after infection, and thus their exclusion did not affect cause-specific death analysis. In addition, 151/557 deaths did not include an ICD-10 code for cause of death; however, records for 127/151 of those case-patients included written cause of death data corresponding to an ICD-10 code that we then added to our dataset.

### Statistical Analyses

We calculated survival time for each patient in person-years from the date of acute WNV onset and ended at either reported date of death or on December 31, 2012. We computed Kaplan-Meier survival curves and stratified by diagnosis of WNND. To identify covariates in our data associated with death, we used Cox proportional hazards regression to determine hazard ratios (HRs) for WNND, age, race, and sex on survival. All variables were coded as nominal variables except age at onset, which was coded as an ordinal variable with 10-year age groupings. We used a backward selection technique to construct a multivariable model. Covariates with p<0.2 were included in multivariable analysis, and variables with p<0.05 were retained in the final model.

Testing of the validity of the proportional hazards assumption by log-log plots and Schoenfeld residuals indicated that the hazard caused by WNND was not constant over time. Therefore, we divided our study population into an acute phase of deaths that occurred within the first 90 days of WNV symptom onset and a convalescent phase of deaths that occurred after the first 90 days of symptom onset. We retained this division for all subsequent analyses because it is standard in chronic death studies. We then developed a Cox model of deaths occurring in the convalescent phase and verified that it met the proportional hazards assumption.

We calculated descriptive statistics to describe acute-phase deaths. We then compared convalescent-phase deaths with those in the Texas population by using SMRs. We obtained all-cause and ICD-10 chapter–specific underlying cause mortality rates for Texas by using the Centers for Disease Control and Prevention WONDER database for calendar years 2002–2012 (*18*). We adjusted for age group, sex, and calendar year and used age group and calendar year as time-varying covariates. We calculated the expected number of deaths in the population by multiplying the person-years by the calendar year–specific and age-specific Texas mortality rates. If a Texas rate was suppressed in the database because of low cell counts, we used US mortality rates.

We then calculated all-cause and cause-specific SMRs and stratified by WNND disease status, age group, and follow-up year. With regards to age grouping, we first stratified by 10-year intervals, then divided the population as >60 and <60 years of age because previous studies found a major increase in severe disease and death risk for persons >60 years of age (*19**–**21*). We computed all statistics and survival curves by using Stata software version 15 (https://www.stata.com).

### Ethics Considerations

This study was approved by the TXDSHS Institutional Review Board (#13–060). Because data transferred from TXDSHS to Baylor College of Medicine for analysis had all identifying information removed, the study was determined exempt by the Baylor College of Medicine Institutional Review Board (H-32097).

## Results

During July 3, 2002–December 31, 2012, a total of 557 (13.4%) deaths occurred among the 4,142 WNV case-patients reported to TXDSHS (Table 1). Time to death ranged from 0 to 3,765 days after onset of symptoms of WNV infection (median 73 days).

**Table 1:** Demographic and clinical characteristics of case-patients with fatal and nonfatal West Nile virus infections, Texas, USA, 2002–2012*

Characteristic	Nonfatal, n = 3,585	Fatal, n = 557	
		Acute phase, n = 289	Convalescent phase, n = 268
Median age at symptom onset, y (range)	52 (0–98)	75 (19–100)	70.5 (17–99)
Sex, %			
M	2,015 (56.2)	175 (60.6)	182 (67.9)
F	1,570 (43.8)	114 (39.4)	86 (32.1)
Race, %			
White, non-Hispanic	2,630 (73.4)	195 (67.5)	182 (67.9)
White, Hispanic	753 (21.0)	64 (22.2)	59 (22.0)
Black	202 (5.6)	30 (10.4)	27 (10.1)
Median no. days until death after symptom onset (range)	NA	19 (0–88)	1,171.5 (92–3,765)
WNND, no. (%)	1,902 (53.1)	267 (92.4)	210 (78.4)
Case counts by year of infection			
2002	203	13	25
2003	598	50	94
2004	137	18	24
2005	154	13	33
2006	279	37	41
2007	209	25	26
2008	57	2	6
2009	103	10	1
2010	76	9	4
2011	22	4	1
2012	1,747	108	13

*Acute indicates that death occurred <90 d after symptom onset after infection with West Nile virus. Convalescent phase indicates that death occurred >90 d after symptom onset. NA, not available; WNND, West Nile neuroinvasive disease.

### Acute-Phase Deaths

**Figure 1:**
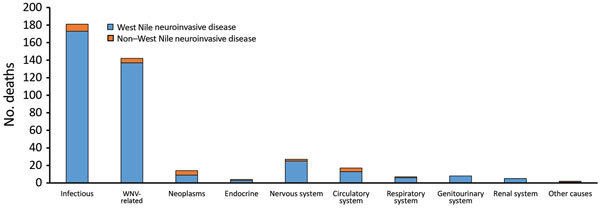
Causes of death in acute cases (within 90 days of WNV disease onset) by condition, Texas, USA, 2002–2012. Most deaths were related to infectious causes (International Classification of Diseases, 10th Revision [ICD10], chapters A00–B99), with a subset of those specifically stating a diagnosis of WNV infection (ICD-10 code A923). Both causes are included in this figure. WNV, West Nile virus.

Approximately half of the deaths (n = 289, 51.9%) occurred during the acute phase after WNV symptom onset. Most (n = 267, 92.4%) of these deaths occurred among WNND case-patients (median age at onset 75 years). For case-patients who died during the acute phase, the most common underlying cause of death was infectious (181 deaths; 62.6%); 142 deaths (49.1%) were specifically attributed to complications from WNV infection (ICD-10 code A923) (Figure 1).

### Convalescent-Phase Deaths

During the convalescent phase, 268 (7.0%) patients died. Most deaths occurred in case-patients with WNND (210/2,112; case-fatality rate 9.9%). This rate was much higher than the case-fatality rate of 3.3% for patients with non-WNND (58/1,741). WNND case-patients contributed 9,573 total years of follow-up time (median 4.37 years/patient), and non-WNND case-patients contributed 5,772 total years (median 0.46 years/patient). The median time elapsed from symptom onset until death was 3.0 years for convalescent-phase WNND patients and 4.1 years for non-WNND case-patients. The disparity in follow-up time between WNND and non-WNND case-patients occurred because a larger proportion of non-WNND case-patients were reported later in the study period. Specifically, 58.1% of non-WNND case-patients were reported in 2012 compared with 35.4% of WNND case-patients.

**Figure 2:**
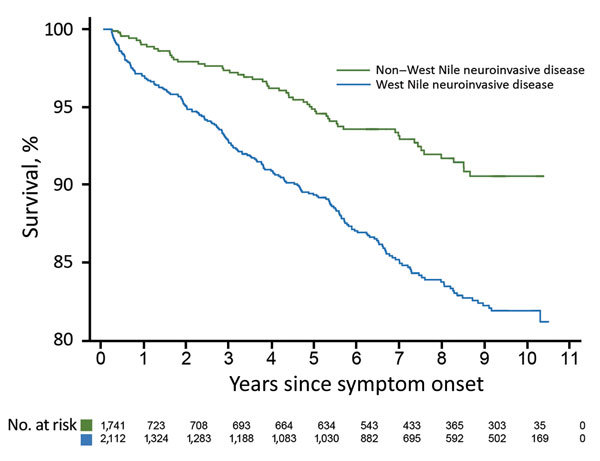
Kaplan-Meier survival curve for case-patients infected with West Nile virus, Texas, USA, 2002–2012, stratified by severity of presenting illness (deaths within 90 days excluded).

We constructed Kaplan-Meier curves comparing deaths for WNND case-patients and deaths for non-WNND case-patients (Figure 2). We found by Cox proportional hazards regression strong associations for death with WNND (HR 1.67, 95% CI 1.24–2.23), age at symptom onset (HR 1.63, 95% CI 1.53–1.75/10-year increase), black race (HR 1.68, 95% CI 1.12–2.52), and male sex (HR 1.42, 95% CI 1.10–1.84).

Among deaths that occurred during the convalescent phase, the incidence of death from any cause for WNND case-patients was much higher per 10,000 person-years (rate 219.4, 95% CI 191.6–251.1) than for non-WNND case-patients(100.5, 95% CI 77.7–129.9). All-cause deaths for WNND case-patients were not increased compared with deaths for the Texas population (SMR 1.13) (Table 2). Conversely, all-cause deaths for non-WNND case-patients were reduced compared with deaths for the Texas population (SMR 0.72, 95% CI 0.55–0.93).

**Table 2:** Causes of convalescent-phase death in persons given a diagnosis of West Nile fever or WNND, Texas, USA, 2002–2012*

ICD-10 code	Cause of death or disorder	WNND				Non-WNND		
		No. deaths observed	No. deaths expected	SMR (95% CI)		No. deaths observed	No. deaths expected	SMR (95% CI)
Any	All cause	210	187	1.13 (0.99–1.29)		58	81.08	0.72 (0.55–0.93)
A00-B99	Infectious	26	5.49	4.74 (3.22–6.94)		3	2.57	1.17 (0.38–3.62)
C00-D48	Neoplasms	33	43.89	0.75 (0.53–1.06)		12	19.06	0.63 (0.36–1.11)
E00-E90	Endocrine	11	8.13	1.35 (0.75–2.44)		3	3.60	0.83 (0.27–2.58)
F00-F99	Mental and behavioral	13	8.96	1.45 (0.84–2.50)		1	3.86	0.26 (0.04–1.84)
G00-G99	Nervous system	11	10.70	1.03 (0.57–1.86)		4	4.51	0.89 (0.33–2.36)
I00-I99	Circulatory system	64	63.44	1.01 (0.79–1.29)		18	26.99	0.67 (0.42–1.06)
J00-J99	Respiratory system	13	19.70	0.66 (0.38–1.14)		7	8.12	0.86 (0.41–1.81)
K00-K93	Digestive system	9	6.82	1.32 (0.69–2.53)		1	3.24	0.31 (0.04–2.19)
N00-N99	Genitourinary system	13	5.40	2.41 (1.40–4.15)		2	2.30	0.87 (0.22–3.47)
N00-N19	Renal disease	11	4.25	2.59 (1.43–4.67)		1	1.81	0.55 (0.08–3.92)
R00-R99	Other	5	2.53	1.97 (0.82–4.74)		2	1.18	1.70 (0.43–6.80)
W00-Y98	External forces	10	6.86	1.46 (0.78–2.71)		3	3.77	0.80 (0.26–2.47)

*Deaths and SMRs were calculated only for cases with information about age available and in which death occurred >90 d after symptom onset. SMR was 1 when there was no increased risk. ICD-10, International Classification of Diseases, 10th Revision; SMR, standardized mortality ratio adjusted for current age, sex, and calendar year; WNND, West Nile neuroinvasive disease.

When we examined cause-specific deaths (Table 2), non-WNND cases were not different for any cause when compared with those in the Texas population. However, among WNND cases, we found increased mortality rates caused by infectious (SMR 4.74) and genitourinary (SMR 2.41) complications. Deaths from infections were caused by primarily WNV complications (ICD-10 code A923, 13/26 deaths), sepsis caused by an unspecified organism (A419, 5/26 deaths), or sequelae of other specified infectious diseases (B948, 4/26 deaths). Deaths from infectious causes occurred at a median of 0.60 years (range 0.25–6.70 years) after symptom onset. Most (11/13) deaths from genitourinary causes were specifically from renal causes (ICD-10 codes N00–N19), which had an increased SMR of 2.59. Renal deaths were caused by chronic kidney disease (ICD-10 code N18, 8/11 deaths), unspecified kidney failure (N19, 2/11 deaths), chronic nephritic syndrome (N039, 1/11 deaths), and acute kidney failure (N179, 1/11 deaths). Deaths caused by renal disease occurred a median of 4.03 years (range 1.36–10.3 years) after symptom onset.

**Figure 3:**
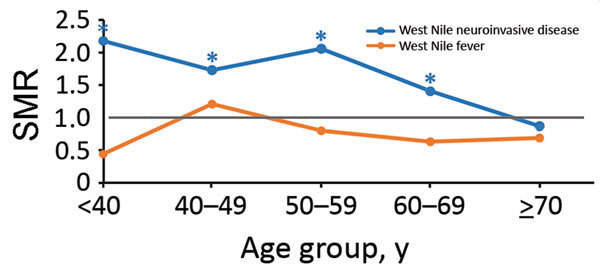
SMRs by age at onset of persons with West Nile virus infection, Texas, USA, 2002–2012. SMRs were adjusted for current age, sex, and calendar year. Deaths and SMRs were calculated only for case-patients with information about age available and in whom death occurred >90 days after symptom onset. SMR = 1 when there is no increased risk. *Indicates where a 95% CI does not include 1. SMR, standardized mortality ratio.

When we stratified by 10-year age groups at onset (Figure 3), we found that for WNND case-patients, all-cause SMRs were considerably increased for age groups <60 years; SMRs were 2.18 (95% CI 1.04–4.56) for persons <40 years of age, 1.73 (95% CI 1.00–2.97) for persons 40–49 years of age, and 2.06 (95% CI 1.46–2.92) for persons 50–59 years of age. All-cause deaths were also increased for persons 60–69 years of age (SMR 1.41, 95% CI 1.06–1.87). For non-WNND case-patients, deaths were lower for persons >70 years of age at symptom onset (SMR 0.69, 95% CI 0.46–0.94).

We divided the WNND case deaths into 2 groups to examine cause-specific deaths for patients <60 years of age and for those >60 of age at disease onset (Table 3). The absolute mortality rate was higher in persons >60 years of age (444.7 deaths/10,000 person-years, 95% CI 380.5–519.8 deaths/10,000 person-years) than in persons <60 years of age (86.4 deaths/10,000 person-years, 95% CI 65.8–113.4 deaths/10,000 person-years). However, all-cause deaths for persons <60 years of age was nearly twice the rate for the entire population of Texas (SMR 1.98). Deaths from infectious causes was increased in both age groups (SMRs 5.33 persons >60 years of age, 4.55 for persons <60 years of age). For patients <60 years of age, we found major increases in deaths from renal (SMR 11.37), mental and behavioral (SMR 6.28), digestive (SMR 3.87), and circulatory (SMR 2.02) causes.

**Table 3:** SMRs for convalescent-phase deaths caused by WNND stratified by age at WNV infection symptom onset, Texas, USA, 2002–2012*

ICD-10 code	Cause of death or disorder	<60 years of age				>60 years of age		
		No. deaths observed	No. deaths expected	SMR (95% CI)		No. deaths observed	No. deaths expected	SMR (95% CI)
Any	All cause	**52**	**26.26**	**1.98 (1.51–2.60)**		158	159.76	0.99 (0.84–1.16)
A00-B99	Infectious	**7**	**1.31**	**5.33 (2.54–11.17)**		**19**	**4.18**	**4.55 (2.90–7.13)**
C00-D48	Neoplasms	8	7.24	1.11 (0.55–2.21)		25	36.73	0.68 (0.46–1.01)
E00-E90	Endocrine	1	1.29	0.78 (0.11–5.51)		10	6.686	1.46 (0.79–2.72)
F00-F99	Mental and behavioral	**2**	**0.32**	**6.28 (1.57–25.12)**		11	8.64	1.27 (0.70–2.30)
G00-G99	Nervous system	2	0.61	3.27 (0.82–13.07)		9	10.09	0.89 (0.46–1.71)
I00-I99	Circulatory system	**14**	**6.94**	**2.02 (1.19–3.41)**		50	56.50	0.88 (0.67–1.17)
J00-J99	Respiratory system	2	1.42	1.40 (0.35–5.62)		11	18.27	0.60 (0.33–1.09)
K00-K93	Digestive system	**7**	**1.81**	**3.87 (1.85–8.12)**		2	5.01	0.40 0.10–1.60)
N00-N99	Genitourinary system	**6**	**0.51**	**11.72 (5.26–26.08)**		7	4.89	1.43 (0.68–3.00)
N00-N19	Renal system	**5**	**0.44**	**11.37 (4.73–27.31)**		6	3.81	1.58 (0.71–3.51)
R00-R99	Other	0	0.42	0 (NA)		5	2.11	2.36 (0.98–5.68)
W00-Y98	External forces	3	2.83	1.06 (0.34–3.29)		7	4.01	1.75 (0.83–3.66)

*Deaths and SMR were calculated only for cases with information about age available and in which death occurred >90 d after symptom onset. SMR was 1 when there was no increased risk. Bold indicates statistical significance. ICD-10, International Classification of Diseases, 10th Revision; NA, not applicable; SMR, standardized mortality ratio adjusted for current age, sex, and calendar year; WNND, West Nile neuroinvasive disease; WNV, West Nile virus.

When we stratified by year of follow-up, we found that SMRs were not increased in any year for non-WNND case-patients; however, we did see a difference among WNND case-patients. We identified a statistically significant increase in SMR for all WNND case-patients in the first year after infection (SMR 1.75, 95% CI 1.32–2.32; p<0.001); this increase continued into years 2 (SMR 1.15) and 3 (SMR 1.33), but not at a statistically significant level.

**Figure 4:**
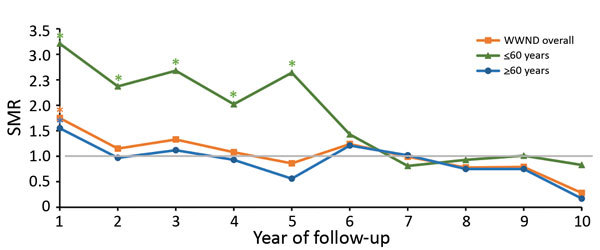
SMRs for case-patients with WNND by year of follow-up stratified by age at symptom onset, Texas, USA, 2002–2012. SMRs were adjusted for current age, sex, and calendar year. Deaths and SMRs were calculated only for case-patients with information about age available and in whom death occurred >90 days after symptom onset. SMR = 1 when there is no increased risk. *Indicates where a 95% CI does not include 1. SMR, standardized mortality ratio; WNND, West Nile neuorinvasive disease.

We then repeated our analysis while stratifying WNND case-patients >60 years of age and those <60 years of age at symptom onset (Figure 4). For case-patients >60 of age, we found increased deaths in the first year after symptom onset (SMR 1.55, 95% CI 1.13–2.14) but not in subsequent years. However, for case-patients <60 years of age, we found increased mortality rates, more than twice the full Texas population rate, until 5 years after WNV infection; deaths were only slightly increased during year 6. Specifically, SMRs were 3.2 (95% CI 1.8–5.8; p = 0.0001) in year 1, 2.4 (95% CI 1.1–5.0; p = 0.002) in year 2, 2.7 (95% CI 1.3–5.4; p = 0.006) in year 3, 2.0 (95% CI 0.9–4.5; p = 0.09) in year 4, and 2.6 (95% CI 1.3–5.3; p = 0.008) in year 5. This increase reached statistical significance in 4 of the 6 years (years 1, 2, 3, and 5). 

## Discussion

We present population-level evidence of excess acute and delayed deaths for a large sample size of case-patients with a history of WNV infection resulting in WNND. Across our full sample, although we did not find increased all-cause mortality rates at a significant level, we did find that deaths from renal and infectious causes for WNND case-patients occurred at a higher rate than would be expected for the full population of Texas. When we stratified by age, we found a major increased risk for all-cause death for case-patients who were <60 years of age at the time of symptom onset. The greatest risks for death in this group were attributed to renal, infectious, digestive, and circulatory causes. 

Our data provide further evidence supporting excess illness and death years after WNV infection resulting from WNND. In addition, we found that reported case-patients with non-WNND had all-cause mortality rates that were lower than the Texas population rate. Our study is unique when compared with previous cohort studies because our large sample size enabled us to explore the progression of deaths for WNV case-patients over a greater period, stratify by age group, stratify by disease presentation, and examine cause-specific deaths (*10**,**11*).

In our study, we found that among persons given a diagnosis of WNND, deaths from renal causes occurred at 2.5 times the rate for the Texas population. Furthermore, when stratifying by age, we found that persons <60 years of age had an 11 times greater risk for dying from renal causes. Deaths caused by kidney disease occurred at median of 4 years after infection, and most were caused by chronic kidney disease (CKD). If one considers that almost half of all of the case-patients in the dataset were infected in 2012, we have comparatively much less follow-up data for later years, when these delayed deaths could occur. It will be useful to conduct a follow-up study on the 2012 cases to determine whether this major finding will continue to be evident.

Increased risk for death from renal causes certainly warrants further investigation. In previous studies that investigated risk factors for WNND, renal disease was not found to be significant (*22*); however, traditional risk factors for CKD (advanced age, hypertension, and diabetes) were found to be associated with WNND. In 1 study, a history of renal disease was found to be associated with an increased risk for death during the acute phase of WNV illness. It is possible that underlying concurrent conditions that increase the risk for WNND in our population could also contribute to death related to renal causes. Unfortunately, our method of only collecting data from death certificates did not enable us to fully examine the medical history. This examination would be critical to further evaluate because previous studies that involved animal models found evidence of persistent WNV infection in renal tissue (*23**–**26*). A study from a Houston WNV cohort isolated WNV RNA from the urine of 5 study participants with a history of WNND (*27*). In a separately published study, Nolan et al. identified CKD in 40% of cohort participants, with history of WNND being the sole independent predictor for CKD as opposed to traditional risk factors (i.e., hypertension, diabetes, or being >65 years of age) (*8*), in comparison with a prevalence of kidney disease in only 8% of patients in this cohort during the acute phase of WNV disease onset (*20*). Although detection of persistent infection of the kidneys in humans has been controversial (*28*), the findings of our study of increased mortality rates from renal causes, particularly CKD, and median renal death occurring 4 years postinfection lends support to studies that have identified renal disease as a possible sequelae of infection with WNV.

WNND case-patients <60 years of age at the time of symptom onset had mortality rates twice the rate expected for the Texas population. These younger patients had excess deaths from renal causes (>11 times) and infectious causes (>5 times) compared with deaths among the general population. In addition, these patients had excess deaths from mental and behavioral, circulatory, and digestive system causes. Conversely, patients >60 years of age at symptom onset had all-cause mortality rates similar to those for the Texas population. However, mortality rates for infectious causes were ≈5 times those for the Texas population. The finding that the convalescent-phase mortality rate was increased for younger patients was unexpected because advanced age is known to be a risk factor for death from infection with WNV during the acute phase of the disease. It will be helpful to expand this study to review these case-patients and determine whether concurrent conditions, particularly hypertension or immunosuppression (*28*), could contribute to the risk for severity of WNV disease and, thereby, increased risk for death.

The pattern through time indicated increased mortality rates for all WNND case-patients for the first year after symptom onset, even after excluding deaths within the first 90 days. WNND case-patients died from infectious causes at >5 times the expected rate, and deaths occurred primarily during the first 2 years: half (13/26) of these deaths were coded as caused by WNV infection, and 4 were attributed to sequelae of other specified infectious disease. Similar results have been reported in cohort studies in Colorado, USA (*10*), and in Israel (*11*), in which increased mortality rates were observed within the first year after infection and there was some evidence of later delayed deaths that did not reach statistical significance.

Non-WNND case-patients unexpectedly had mortality rates lower than those for the Texas population. The reasons for this finding are not clear. However, the population that seeks care resulting in a non-WNND diagnosis might be healthier than the general Texas population. In our study, half of the non-WNND case-patients were given a diagnosis during 2012, which was during an outbreak in Texas that was larger than any previously recorded and well publicized in the media (*21*). Because of increased awareness, more healthy persons might have sought care resulting in a diagnosis of non-WNND.

Our study had several limitations. First, because of our register-based method, we were unable to assess previous concurrent conditions in the study population. Major known contributory concurrent conditions from previous mortality studies include dementia, cardiovascular disease, hepatic disease, immunodeficiency, autoimmune disease, use of tobacco, alcoholism, and intubation during acute WNV illness (*10**,**11*). The increased mortality rate we observed in younger patients might be caused by this population being less healthy than the underlying Texas population and possessing >1 of these risk factors. This hypothesis is supported by the increased mortality rate for infectious, circulatory, and digestive system causes among younger case-patients. However, for these younger case-patients, deaths from renal causes were most increased in comparison with those for the Texas population, and deaths from infectious causes were primarily the result of direct WNV infection or sequelae of WNV infection. Moreover, when examined within the context of the whole study population, we found that deaths from renal and infectious causes remained higher than those for the Texas population, although all other causes were not. Analysis of contributing cause of death data might enable greater assessment of concurrent conditions for deaths caused by WNV.

Second, our study might have undercounted deaths in our population because of effects of interstate and international migration. We were only able to obtain death certificate data for WNV case-patients who died in Texas. Thus, we are likely underestimating the SMRs in our population, given substantial levels of migration in and out of Texas. Finally, our study has limited generalizability: <1% of WNV infections result in WNND, and most of these patients are elderly (>60 years of age) (*2*). We found mortality rates to be most increased in younger patients with WNND and found no evidence of deaths for case-patients with a history of WNF.

In conclusion, arboviruses continue to be an emerging global threat, and defining the long-term consequences of WNV is critical. We present population-level evidence for increased risk for death, particularly for patients <60 of age who have a history of WNND. For these patients, excess deaths were related to infectious and renal causes, and excess all-cause deaths were evident for <5 years after onset of symptoms. No specific treatment is available for WNV infection; therefore, prevention of infection is key, either through education efforts to encourage avoiding mosquito bites, comprehensive mosquito surveillance and control, or a greater emphasis on developing an effective vaccine. WNND patients should be closely followed by clinicians to prevent future health problems.
