# Correction: Vol. 25, No. 2

**DOI:** 10.3201/eid2505.C22505

**Published:** 2019-05

**Keywords:** errata, erratum, correction

The number of cases of West Nile neuroinvasive disease was listed incorrectly in the abstract of Acute and Delayed Deaths after West Nile Virus Infection, Texas, USA, 2002–2012 (D.C.E. Philpott et al.). The article has been corrected online (https://wwwnc.cdc.gov/eid/article/25/2/18-1250_article).

